# How to measure metacognition

**DOI:** 10.3389/fnhum.2014.00443

**Published:** 2014-07-15

**Authors:** Stephen M. Fleming, Hakwan C. Lau

**Affiliations:** ^1^Department of Experimental Psychology, University of OxfordOxford, UK; ^2^Center for Neural Science, New York UniversityNew York, NY, USA; ^3^Department of Psychology, Columbia UniversityNew York, NY, USA; ^4^Department of Psychology, University of California, Los AngelesLos Angeles, CA, USA

**Keywords:** metacognition, confidence, signal detection theory, consciousness, probability judgment

## Abstract

The ability to recognize one's own successful cognitive processing, in e.g., perceptual or memory tasks, is often referred to as metacognition. How should we quantitatively measure such ability? Here we focus on a class of measures that assess the correspondence between trial-by-trial accuracy and one's own confidence. In general, for healthy subjects endowed with metacognitive sensitivity, when one is confident, one is more likely to be correct. Thus, the degree of association between accuracy and confidence can be taken as a quantitative measure of metacognition. However, many studies use a statistical correlation coefficient (e.g., Pearson's *r*) or its variant to assess this degree of association, and such measures are susceptible to undesirable influences from factors such as response biases. Here we review other measures based on signal detection theory and receiver operating characteristics (ROC) analysis that are “bias free,” and relate these quantities to the calibration and discrimination measures developed in the probability estimation literature. We go on to distinguish between the related concepts of metacognitive *bias* (a difference in subjective confidence despite basic task performance remaining constant), metacognitive *sensitivity* (how good one is at distinguishing between one's own correct and incorrect judgments) and metacognitive *efficiency* (a subject's level of metacognitive sensitivity given a certain level of task performance). Finally, we discuss how these three concepts pose interesting questions for the study of metacognition and conscious awareness.

## Introduction

Early cognitive psychologists were interested in how well people could assess or monitor their own knowledge, and asking for confidence ratings was one of the mainstays of psychophysical analysis (Peirce and Jastrow, 1885). For example, Henmon (1911) summarized his results as follows: “While there is a positive correlation on the whole between degree of confidence and accuracy the degree of confidence is not a reliable index of accuracy.” This statement is largely supported by more recent research in the field of metacognition in a variety of domains from memory to perception and decision-making: subjects have some metacognitive sensitivity, but it is often subject to error (Nelson and Narens, 1990; Metcalfe and Shimamura, 1996). The determinants of metacognitive sensitivity is an active topic of investigation that has been reviewed at length elsewhere (e.g., Koriat, 2007; Fleming and Dolan, 2012). Here we are concerned with the best approach to measure metacognition, a topic on which there remains substantial confusion and heterogeneity of approach.

**Figure 1:**
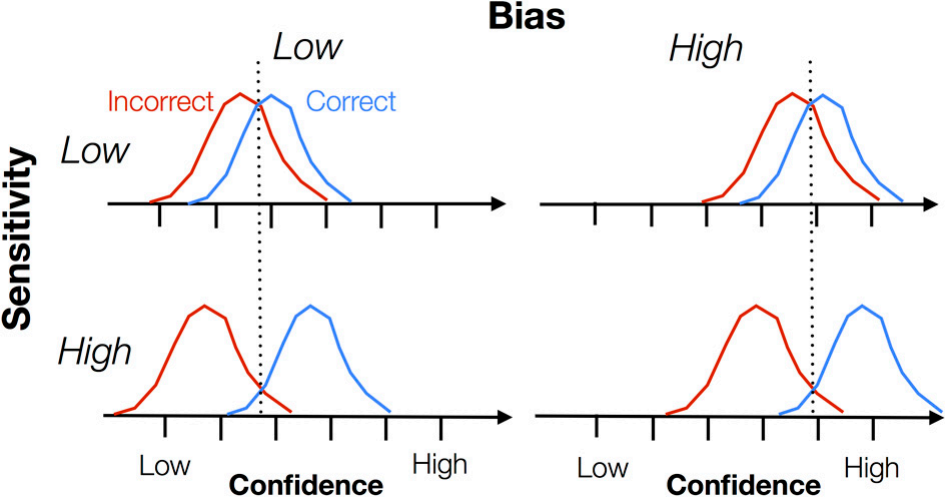
**Schematic showing the theoretical dissociation between metacognitive sensitivity and bias**. Each graph shows a hypothetical probability density of confidence ratings for correct and incorrect trials, with confidence increasing from left to right along each x-axis. Metacognitive sensitivity is the separation between the distributions—the extent to which confidence discriminates between correct and incorrect trials. Metacognitive bias is the overall level of confidence expressed, independent of whether the trial is correct or incorrect. Note that this is a cartoon schematic and we do not mean to imply any parametric form for these “Type 2” signal detection theoretic distributions. Indeed, as shown by Galvin et al. (2003), these distributions are unlikely to be Gaussian.

From the outset, it is important to distinguish two aspects, namely sensitivity and bias. Metacognitive *sensitivity* is also known as metacognitive accuracy, type 2 sensitivity, discrimination, reliability, or the confidence-accuracy correlation. Metacognitive *bias* is also known as type 2 bias, over- or underconfidence or calibration. In Figure 1 we illustrate the difference between these two constructs. Each panel shows a cartoon density of confidence ratings separately for correct and incorrect trials on an arbitrary task (e.g., a perceptual discrimination). Intuitively, when these distributions are well separated, the subject is able to discriminate good and bad task performance using the confidence scale, and can be assigned a high degree of metacognitive sensitivity. However, note that bias “rides on top of” any measure of sensitivity. A subject might have high overall confidence but poor metacognitive sensitivity if the correct/error distributions are not separable. Both sensitivity and bias are important features of metacognitive judgments, but they are often conflated when interpreting data. In this paper we outline behavioral measures that are able to separately quantify sensitivity and bias.

A second important feature of metacognitive measures is that sensitivity is often affected by task performance itself—in other words, the same individual will appear to have greater metacognitive sensitivity on an easy task compared to a hard task. In contrast, it is reasonable to assume that an individual might have a particular level of metacognitive *efficiency* in a domain such as memory or decision-making that is independent of different levels of task performance. Nelson (1984) emphasized this desirable property of a measure of metacognition when he wrote that “there should not be a built-in relation between [a measure of] feeling-of-knowing accuracy and overall recognition,” thus providing for the “logical independence of metacognitive ability… and objective memory ability” (Nelson, 1984; p. 111). The question is then how to distil a measure of metacognitive efficiency from behavioral data. We highlight recent progress on this issue.

We note there are a variety of methods for eliciting metacognitive judgments (e.g., wagering, scoring rules, confidence scales, awareness ratings) across different domains that have been discussed at length elsewhere (Keren, 1991; Hollard et al., 2010; Sandberg et al., 2010; Fleming and Dolan, 2012). Our focus here is on quantifying metacognition once a judgment has been elicited.

## Measures of metacognitive sensitivity

A useful starting point for all the measures of metacognitive sensitivity that follow is the 2 × 2 confidence-accuracy table (Table 1). This table simply counts the number of high confidence ratings assigned to correct and incorrect judgments, and similarly for low confidence ratings. Intuitively, above-chance metacognitive sensitivity is found when correct trials are endorsed with high confidence to a greater degree than incorrect trials^1^. Readers with a background in signal detection theory (SDT) will immediately see the connection between Table 1 and standard, “type 1” SDT (Green and Swets, 1966). In type 1 SDT, the relevant joint probability distribution is *P*(*response, stimulus*)—parameters of this distribution such as *d*′ are concerned with how effectively an organism can discriminate objective states of the world. In contrast, Table 1 has been dubbed the “type 2” SDT table (Clarke et al., 1959), as the confidence ratings are conditioned on the observer's responses (correct or incorrect), not on the objective state of the world. All measures of metacognitive sensitivity can be reduced to operations on this joint probability distribution *P*(*confidence*, *accuracy*) (see Mason, 2003, for a mathematical treatment).

**Table 1:** **Classification of responses within type 2 signal detection theory**.

**Type I decision**	**High confidence**	**Low confidence**
Correct	Type 2 hit (H2)	Type 2 miss (M2)
Incorrect	Type 2 false alarm (FA2)	Type 2 correct rejection (CR2)

In the discussion that follows we assume that stimulus strength or task difficulty is held roughly constant. In such a design, fluctuations in accuracy and confidence can be attributed to noise internal to the observer, rather than external changes in signal strength. This “method of constant stimuli” is appropriate for fitting signal detection theoretic models, but it also rules out other potentially interesting experimental questions, such as how behavior and confidence change with stimulus strength. In the section Psychometric Function Measures we discuss approaches to measuring metacognitive sensitivity in designs such as these.

### Correlation measures

The simplest measure of association between the rows and columns of Table 1 is the phi (ϕ) correlation. In essence, phi is the standard Pearson *r* correlation between accuracy and confidence over trials. That is, if we code correct responses as 1's, and incorrect responses as 0's, accuracy over trials forms a vector, e.g., [0 1 1 0 0 1]. And if we code high confidence as 1, and low confidence as 0, we can likewise form a vector of the same length (number of trials). The Pearson *r* correlation between these two vectors defines the “phi” coefficient. A related and very common measure of metacognitive sensitivity, at least in the memory literature, is the Goodman–Kruskall gamma coefficient, *G* (Goodman and Kruskal, 1954; Nelson, 1984). In a classic paper, Nelson (1984) advocated *G* as a measure of metacognitive sensitivity that does not make the distributional assumptions of SDT.

*G* can be easily expanded to handle designs in which confidence is made using a rating scale rather than a dichotomous high/low design (Gonzalez and Nelson, 1996). Though popular, as measures of metacognitive sensitivity both phi and gamma correlations have a number of problems. The most prominent is the fact that both can be “contaminated” by metacognitive bias. That is, for subjects with a high or low tendency to give high confidence ratings overall, their phi correlation will be altered (Nelson, 1984)^2^. Intuitively one can consider the extreme cases where subjects perform a task near threshold (i.e., between ceiling and chance performance), but rate every trial as low confidence, not because of a lack of ability to introspect, but because of an overly shy or humble personality. In such a case, the correspondence between confidence and accuracy is constrained by bias. In an extensive simulation study, Masson and Rotello (2009) showed that *G* was similarly sensitive to the tendency to use higher or lower confidence ratings (bias), and that this may lead to erroneous conclusions, such as interpreting a difference in *G* between groups as reflecting a true underlying difference in metacognitive sensitivity despite possible differences in bias.

### Type 2 *d*′

A standard way to remove the influence of bias in an estimation of sensitivity is to apply SDT (Green and Swets, 1966). In the case of type 1 detection tasks, overall percentage correct is “contaminated” by the subject's bias, i.e., the propensity to say “yes” overall. To remove this influence of bias, researchers often estimate *d*′ based on the hit rate and false alarm rate, which (assuming equal-variance Gaussian distributions for internal signal strength) is mathematically independent of bias. That is, given a constant underlying sensitivity to detect the signal, estimated *d*′ will be constant given different biases.

There have been several evaluations of this approach to characterize metacognitive sensitivity (Clarke et al., 1959; Lachman et al., 1979; Ferrell and McGoey, 1980; Nelson, 1984; Kunimoto et al., 2001; Higham, 2007; Higham et al., 2009), where type 2 hit rate is defined as the proportion of trials in which subjects reported high confidence given their responses were correct (H2 in Table 1), and type 2 false alarm rate is defined as the proportion of trials in which subjects reported high confidence given their responses were incorrect (FA2 in Table 1). Type 2 *d*′ = *z*(H2) − *z*(FA2), where *z* is the inverse of the cumulative normal distribution function^3^. Theoretically, then, by using standard SDT, type 2 *d*′ is argued to be independent from metacognitive bias (the overall propensity to give high confidence responses).

However, type 2 *d*′ turns out to be problematic because SDT assumes that the distribution of internal signals for “correct” and “incorrect” trials are Gaussian with equal variances. While this assumption is usually more or less acceptable at the type 1 level (especially for 2-alternative forced-choice tasks), it is highly problematic for type 2 analysis. Galvin et al. (2003) showed that these distributions are of different variance and highly non-Gaussian if the equal variance assumption holds at the type 1 level. Using simulation data, Evans and Azzopardi (2007) showed that this leads to the type 2 *d*′ measure proposed by Kunimoto et al. (2001) being confounded by changes in metacognitive bias.

### Type 2 ROC analysis

Because the standard parametric signal detection approach is problematic for type 2 analysis, one solution is to apply a non-parametric analysis that is free from the equal-variance Gaussian assumption. In type 1 SDT this is standardly achieved via ROC (receiver operating characteristic) analysis, in which data are obtained from multiple response criteria. For example, if the payoffs for making a hit and false alarm are systematically altered, it is possible to systematically induce more conservative or liberal criteria. For each criterion, hit rate and false alarm rate can be calculated. These are plotted as individual points on the ROC plot—hit rate is plotted on the vertical axis and false alarm rate on the horizontal axis. With multiple criteria we have multiple points, and the curve that passes through these different points is the ROC curve. If the area under the ROC is 0.5, performance is at chance. Higher area under ROC (AUROC) indicates higher sensitivity.

**Figure 2:**
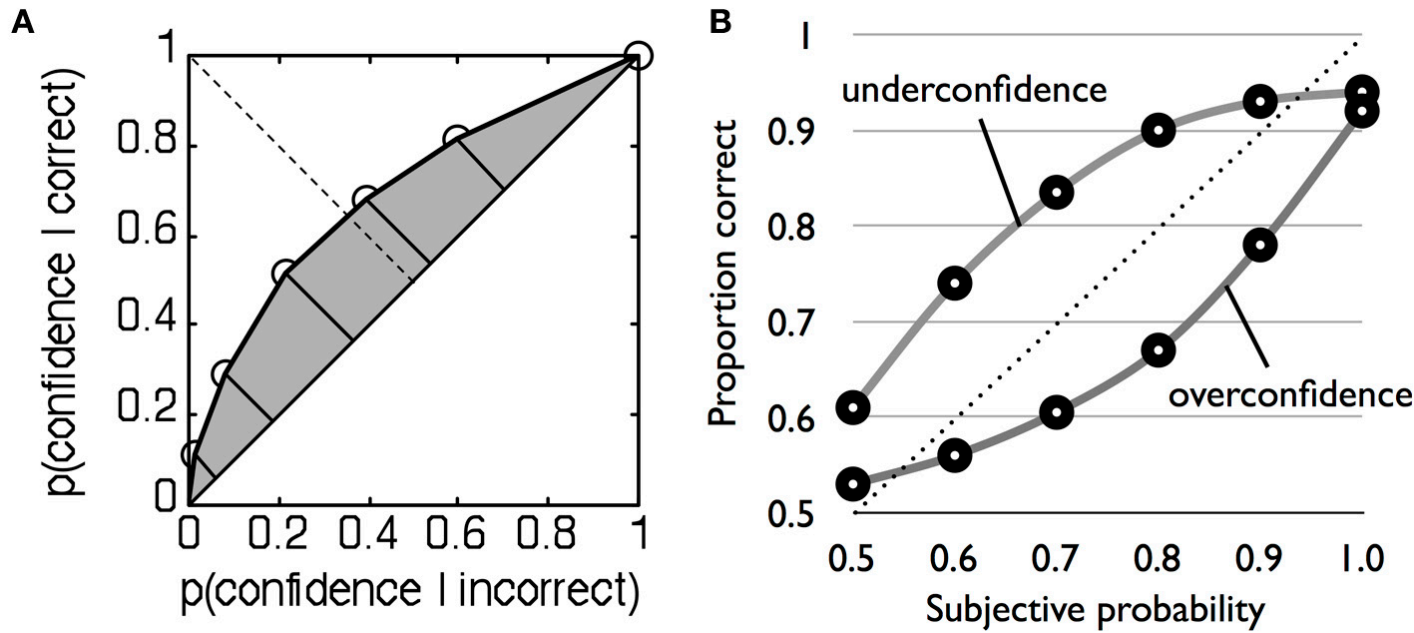
**(A)** Example type 2 ROC function for a single subject. Each point plots the type 2 false alarm rate on the x-axis against the type 2 hit rate on the y-axis for a given confidence criterion. The shaded area under the curve indexes metacognitive sensitivity. **(B)** Example underconfident and overconfident probability calibration curves, modified after Harvey (1997).

Because this method is non-parametric, it does not depend on rigid assumptions about the nature of the underlying distributions and can similarly be applied to type 2 data. Recall that type 2 hit rate is simply the proportion of high confidence trials when the subject is correct, and type 2 false alarm rate is the proportion of high confidence trials when the subject is incorrect (Table 1). For two levels of confidence there is thus one criterion, and one pair of type 2 hit and false alarm rates. However, with multiple confidence ratings it is possible to construct the full type 2 ROC by treating each confidence level as a criterion that separates high from low confidence (Clarke et al., 1959; Galvin et al., 2003; Benjamin and Diaz, 2008). For instance, we start with a liberal criterion that assigns low confidence = 1 and high confidence = 2–4, then a higher criterion that assigns low confidence = 1 and 2 and high confidence = 3 and 4, and so on. For each split of the data, hit and false alarm rate pairs are calculated and plotted to obtain a type 2 ROC curve (Figure 2A). The area under the type 2 ROC curve (AUROC2) can then be used as a measure of metacognitive sensitivity (in the Supplementary Material we provide Matlab code for calculating AUROC2 from rating data). This method is more advantageous than the gamma and phi correlations because it is bias-free (i.e., it is theoretically uninfluenced by the overall propensity of the subject to say high confidence) and in contrast to type 2 *d*′ does not make parametric assumptions that are known to be false.

In summary, therefore, despite their intuitive appeal, simple measures of association such as the phi correlation and gamma do not separate metacognitive sensitivity from bias. Non-parametric methods such as AUROC2 provide bias-free measures of sensitivity. However, a further complication when studying metacognitive sensitivity is that the measures reviewed above are also affected by task performance. For instance, Galvin et al. (2003) showed mathematically that AUROC2 is affected by both type 1 *d*′ and type 1 criterion placement, a conclusion supported by experimental manipulation (Higham et al., 2009). In other words, a change in task performance is expected, *a priori*, to lead to changes in AUROC2, despite the subject's endogenous metacognitive “efficiency” remaining unchanged. One approach to dealing with this confound is to use psychophysical techniques to control for differences in performance and then calculate AUROC2 (e.g., Fleming et al., 2010). An alternative approach is to explicitly model the connection between performance and metacognition.

### Model-based approaches

The recently developed meta-*d*′ measure (Maniscalco and Lau, 2012, 2014) exploits the fact that given Gaussian variance assumptions at the type 1 level, the shapes of the type 2 distributions are known even if they are not themselves Gaussian (Galvin et al., 2003). Theoretically therefore, ideal, maximum type 2 performance is constrained by one's type 1 performance. Intuitively, one can again consider the extreme cases. Imagine a subject is performing a two-choice discrimination task completely at chance. Half of their trials are correct and half are incorrect due to chance responding despite zero type 1 sensitivity. To introspectively distinguish between correct and incorrect trials would be impossible, because the correct trials are flukes. Thus, when type 1 sensitivity is zero, type 2 sensitivity (metacognitive sensitivity) should also be so. This dependency places strong constraints on a measure of metacognitive sensitivity.

Specifically, given a particular type 1 variance structure and bias, the form of the type 2 ROC is completely determined (Galvin et al., 2003). We can thus create a family of type 2 ROC curves, each of which will correspond to an underlying type 1 sensitivity assuming that the subject is metacognitively ideal (i.e., has maximal type 2 sensitivity given a certain type 1 sensitivity). Because such a family of type 2 ROC curves are all non-overlapping (Galvin et al., 2003), we can determine the curve from this family with just a single point, i.e., a single criterion. With this, we can obtain, given the subject's *actual* type 2 performance data, the underlying type 1 sensitivity that we expect if the subject is ideal is placing their confidence ratings. We label the underlying type 1 sensitivity of this ideal observer meta-*d'*. Because meta-*d'* is in units of type 1 *d*′, we can think of it as the sensory evidence available for metacognition in signal-to-noise ratio units, just as type 1 *d*′ is the sensory evidence available for decision-making in signal-to-noise ratio units. Among currently available methods, we think meta-*d*′ is the best measure of metacognitive sensitivity, and it is quickly gaining popularity (e.g., Baird et al., 2013; Charles et al., 2013; Lee et al., 2013; McCurdy et al., 2013). Barrett et al. (2013) have conducted extensive normative tests of meta-*d*′, finding that it is robust to changes in bias and that it recovers simulated changes in metacognitive sensitivity (see also Maniscalco and Lau, 2014). Matlab code for fitting meta-*d*′ to rating data is available at http://www.columbia.edu/~bsm2105/type2sdt/.

One major advantage of meta-*d*′ over AUROC2 is its ease of interpretation and its elegant control over the influence of performance on metacognitive sensitivity. Specifically, because meta-*d*′ is in the same units as (type 1) *d*′, the two can be directly compared. Therefore, for a metacognitively ideal observer (a person who is rating confidence using the maximum possible metacognitive sensitivity), meta-*d*′ should equal *d*′. If meta-*d*′ < *d*′, metacognitive sensitivity is suboptimal within the SDT framework. We can therefore define metacognitive *efficiency* as the value of meta-*d*′ relative to *d*′, or meta-*d*′/*d*′. A meta-*d*′/*d*′ value of 1 indicates a theoretically ideal value of metacognitive efficiency. A value of 0.7 would indicate 70% metacognitive efficiency (30% of the sensory evidence available for the decision is lost when making metacognitive judgments), and so on. A closely related measure is the difference between meta-*d*′ and *d*′, i.e., meta-*d*′ − *d*′ (Rounis et al., 2010). One practical reason for using meta-*d*′ − *d*′ rather than meta-*d*′/*d*′ is that the latter is a ratio, and when the denominator (*d*′) is small, meta-*d*′/*d*′ can give rather extreme values which may undermine power in a group statistical analysis. However, this problem can also be addressed by taking log of meta- *d*′/*d*′, as is often done to correct for the non-normality of ratio measures (Howell, 2009). Toward the end of this article we explore the implications of this metacognitive efficiency construct for a psychology of metacognition.

The meta-*d*′ approach is based on an ideal observer model of the link between type 1 and type 2 SDT, using this as a benchmark against which to compare subjects' metacognitive efficiency. However, meta-*d*′ is unable to discriminate between different causes of a change in metacognitive efficiency. In particular, like standard SDT, meta-*d*′ is unable to dissociate trial-to-trial variability in the placement of confidence criteria from additional noise in the evidence used to make the confidence rating—both manifest as a decrease in metacognitive efficiency.

A similar bias-free approach to modeling metacognitive accuracy is the “Stochastic Detection and Retrieval Model” (SDRM) introduced by Jang et al. (2012). The SDRM not only measures metacognitive accuracy, but is also able to model different potential causes of metacognitive inaccuracy. The core of the model assumes two samplings of “evidence” per stimulus, one leading to a first-order behavior, such as memory retrieval, and the other leading to a confidence rating. These samples are distinct but drawn from a bivariate distribution with correlation parameter ρ. This variable correlation naturally accounts for dissociations between confidence and accuracy. For instance, if the samples are highly correlated, the subject will tend to be confident when behavioral performance is high, and less confident when behavioral performance is low. The SDRM additionally models noise in the confidence rating process itself through variability in the setting of confidence criteria from trial to trial. SDRM was originally developed to account for confidence in free recall involving a single class of items, but it can be naturally extended to two choice cases such as perceptual or mnemonic decisions. By modeling these two separate sources of variability, SDRM is able to unpack potential causes of a decrease in metacognitive efficiency. However, SDRM requires considerable interpretation of parameter fits to draw conclusions about underlying metacognitive processes, and meta-*d*′ may prove simpler to calculate and work with for many empirical applications.

### Metacognitive bias

Metacognitive bias is the tendency to give high confidence ratings, all else being equal. The simplest of such measures is the percentage of high confidence trials (i.e., the marginal proportion of high confidence judgments in Table 1, averaging over correct and incorrect trials), or the average confidence rating over trials. In standard type 1 SDT, a more liberal metacognitive bias corresponds to squeezing the flanking confidence-rating criteria toward the central decision criterion such that more area under both stimulus distributions falls beyond the “high confidence” criteria.

A more liberal metacognitive bias leads to different patterns of responding depending on how confidence is elicited. If confidence is elicited secondary to a decision about options “A” or “B,” squeezing the confidence criteria will lead to an overall increase in confidence, regardless of previous response. However, confidence is often elicited alongside the decision itself, using a scale such as 1 = sure “A” to 6 = sure “B,” where ratings 3 and 4 indicate low confidence “A” and “B,” respectively. A more liberal metacognitive bias in this case would lead to an increased use of the extremes of the scale (1 and 6) and a decreased use of the middle of the scale (3 and 4).

### Psychometric function measures

The methods for measuring metacognitive sensitivity we have discussed above assume data is obtained using a constant level of task difficulty or stimulus strength, equivalent to obtaining a measure of *d*′ in standard psychophysics. If a continuous range of stimulus difficulties are available, such as when a full psychometric function is estimated, it is of course possible to apply the same methods to each level of stimulus strength independently. An alternative approach is to compute an aggregate measure of metacognitive sensitivity as the difference in slope between psychometric functions constructed from high and low confidence trials (e.g., De Martino et al., 2013; de Gardelle and Mamassian, 2014). The extent to which the slope becomes steeper (more accurate) under high compared to low confidence is a measure of metacognitive sensitivity. However, this method may not be bias-free, or account for individual differences in task performance, as discussed above.

### Discrepancy measures

We close this section by pointing out that some researchers have used “one-shot” discrepancy measures to quantify metacognition. For instance, if we ask someone how good their memory is on a scale of 1–10, we obtain a rating that we can then compare to memory performance on a variety of tasks. This discrepancy score approach is often used in the clinical literature (e.g., Schmitz et al., 2006) and in social psychology (e.g., Kruger and Dunning, 1999) to quantify metacognitive skill or “insight.” It is hopefully clear from the preceding sections that if one only has access to a single rating of performance, it is not possible to tease apart bias from sensitivity, nor measure efficiency. To continue with the memory example, a large discrepancy score may be due to a reluctance to rate oneself as performing poorly (metacognitive bias), or a true blindness to one's memory performance (metacognitive sensitivity). In contrast, by collecting trial-by-trial measures of performance and metacognitive judgments we can build up a picture of an individual's bias, sensitivity and efficiency in a particular domain.

## Judgments of probability

Metacognitive confidence can be formalized as a probability judgment directed toward one's own actions—the probability of a previous judgment being correct. There is a rich literature on the correspondence between subjective judgments of probability and the reality to which those judgments correspond. For example, a weather forecaster may make several predictions of the chance of rain throughout the year; if the average prediction (e.g., 60%) ends up matching the frequency of rainy days in the long run we can say that the forecaster is well calibrated. In this framework metacognition has a normative interpretation as the accuracy of a probability judgment about one's own performance. We do not aim to cover the literature on probability judgments here; instead we refer the reader to several comprehensive reviews (Lichtenstein et al., 1982; Keren, 1991; Harvey, 1997; Moore and Healy, 2008). Instead we highlight some developments in the judgment and decision-making literature that directly bear on the measurement of metacognition.

There are two general classes of probability judgment problem. Discrete cases refer to probabilities assigned to particular statements, such as “the correct answer is A” or “it will rain tomorrow.” Continuous cases are where the assessor provides a confidence interval or some other indication of their uncertainty in a quantity such as the distance from London to Manchester. While the accuracy of continuous judgments is also of interest, our focus here is on discrete judgments, as they provide the clearest connection to the metacognition measures reviewed above. For example, in a 2AFC task with stimulus class *d* and response *a*, an ideal observer should base their confidence on the quantity *P*(*d* = *a*).

An advantage of couching metacognitive judgments in a probability framework is that a meaningful measure of bias can be elicited. In other words, while a confidence rating of “4” does not mean much outside of the context of the experiment, a probability rating of 0.7 can be checked against the objective likelihood of occurrence of the event in the environment; i.e., the probability of being correct for a given confidence level. Moreover, probability judgments can be compared against quantities derived from probabilistic models of confidence (e.g., Kepecs and Mainen, 2012).

### Quantifying the accuracy of probability judgments

The judgment and decision-making literature has independently developed indices of probability accuracy similar to *G* and meta- *d*′ in the metacognition literature. For example, following Harvey (1997), a “probability score” (PS) is the squared difference between the probability rating *f* and its actual occurrence *c* (where *c* = 1 or 0 for binary events, such as correct or incorrect judgments):

$$PS={\left(f-c\right)}^{2}$$

The mean value of the PS averaged across estimates is known as the Brier score (Brier, 1950). As the PS is an “error” score, a lower value of *PS* is better. The Brier score is analogous to the phi coefficient discussed above.

The decomposition of the Brier score into its component parts may be of particular interest to metacognition researchers. Particularly, one can decompose the Brier score into the following components (Murphy, 1973):

PS = O + C −R

where *O* is the “outcome index” and reflects the variance of the outcome event *c*: *O* = *c*(1 − *c*); *C* is “calibration,” the goodness of fit between probability assessments and the corresponding proportion of correct responses; and *R* is “resolution,” the variance of the probability assessments. Note that in studies of metacognitive confidence in decision-making, memory, etc., the outcome event is simply the performance of the subject. In other words, when performance is near chance, the variance of the outcomes—corrects and errors—is maximal, and *O* will be high. In contrast, when performance is near ceiling, *O* is low. This decomposition therefore echoes the SDT-based analysis discussed above, and accordingly both reach the same conclusion: simple correlation measures between probabilities/confidence and outcomes/performance are themselves influenced by task performance. Just as efforts have been made to correct measures of metacognitive sensitivity for differences in performance and bias, similar concerns led to the development of bias-free measures of discrimination. In particular, Yaniv et al. (1991) describe an “adjusted normalized discrimination index” (ANDI) that achieves such control.

Calibration (*C*) is defined as:

$$C=\frac{1}{N}\sum_{j=1}^{J}N_{j}{\left(f_{j}-\bar{c_{j}}\;\right)}^{2}$$

where *j* indexes each probability category. Calibration quantifies the discrepancy between the mean performance level in a category (e.g., 60%) and its associated rating (e.g., 80%), with a lower discrepancy giving a better PS. A calibration curve is constructed by plotting the relative frequency of correct answers in each probability judgment category (e.g., 50–60%) against the mean probability rating for the category (e.g., 55%) (Figure 2B). A typical finding is that observers are overconfident (Lichtenstein et al., 1982)—probability judgments are greater than mean % correct.

Resolution is a measure of the variance of the probability assessments, measuring the extent to which correct and incorrect answers are assigned to different probability categories:

$$R=\frac{1}{N}\sum_{j=1}^{J}N_{j}{\left(\bar{c_{j}}\;-\bar{c}\;\right)}^{2}$$

As *R* is subtracted from the other terms in the PS, a larger variance is better, reflecting the observer's ability to place correct and incorrect judgments in distinct probability categories.

Both calibration and resolution contribute to the overall “accuracy” of probability judgments. To illustrate this, consider the following contrived example. In a general knowledge task, a subject rates each correct judgment as 90% likely to be correct, and each error as 80% likely to be correct. Her objective mean performance level is 60%. She is poorly calibrated, in the sense that the mean subjective probability of being correct outstrips her actual performance. But she displays good resolution for discriminating correct from incorrect trials using distinct levels of the probability scale (although this resolution could be even higher if she chose even more diverse ratings). This example raises important questions as to the psychological processes that permit metacognitive discrimination of internal states (e.g., resolution, or sensitivity) and the mapping of these discriminations onto a probability or confidence scale (calibration; e.g., Ferrell and McGoey, 1980). The learning of this mapping, and how it may lead to changes in metacognition, has received relatively little attention.

## Implications of bias, sensitivity, and efficiency for a psychology of metacognition

The psychological study of metacognition has been interested in elucidating the determinants and impact of metacognitive sensitivity. For instance, in a classic example, judgments of learning (JOLs) show better sensitivity when the delay between initial learning and JOL is increased (Nelson and Dunlosky, 1991), presumably due to delayed JOLs recruiting relevant diagnostic information from long-term memory. However, many of these “classic” findings in the metacognition rely on measures such as *G* (Rhodes and Tauber, 2011) that may be confounded by bias and performance effects (although see Jang et al., 2012). We strongly urge the application of bias-free measures of metacognitive sensitivity reviewed above in future studies.

More generally, we believe it is important to distinguish between metacognitive sensitivity and efficiency. To recap, metacognitive sensitivity is the ability to discriminate correct from incorrect judgments; signal detection theoretic analysis shows that metacognitive sensitivity scales with task performance. In contrast, metacognitive efficiency is measured *relative* to a particular performance level. Efficiency measures have several possible applications. First, we may want to compare metacognitive efficiency across domains in which it is not possible to match performance levels. For instance, it is possible to quantify metacognitive efficiency on visual and memory tasks to elucidate their respective neural correlates (Baird et al., 2013; McCurdy et al., 2013). Second, it is of interest to determine whether different subject groups, such as patients and controls (David et al., 2012) or older vs. younger adults (Souchay et al., 2000), exhibit differential metacognitive efficiency after taking into account differences in task performance. For example, Weil et al. (2013) showed that metacognitive efficiency increases during adolescence, consistent with the maturation of prefrontal regions thought to underpin metacognition (Fleming and Dolan, 2012). Finally, it will be of particular interest to compare metacognitive efficiency across different animal species. Several studies have established the presence of metacognitive *sensitivity* in some non-human animals (Hampton, 2001; Kornell et al., 2007; Middlebrooks and Sommer, 2011; Kepecs and Mainen, 2012). However, it is unknown whether other species such as macaque monkeys have levels of metacognitive *efficiency* similar to those seen in humans.

Finally, the influence of performance, or skill, on efficiency itself is of interest. In a highly cited paper, Kruger and Dunning (1999) report a series of experiments in which the worst-performing subjects on a variety of tests showed a bigger discrepancy between actual performance and a one-shot rating than the better performers. The authors concluded that “those with limited knowledge in a domain suffer a dual burden: Not only do they reach mistaken conclusions and make regrettable errors, but their incompetence robs them of the ability to realize it” (p. 1132). Notably the Dunning–Kruger effect has two distinct interpretations in terms of sensitivity and efficiency. On the one hand the effect is a direct consequence of metacognitive sensitivity being determined by type 1 *d*′. In other words, it would be strange (based on the ideal observer model) if worse performing subjects didn't make noisier ratings. On the other hand, it is possible that skill in a domain and metacognitive efficiency share resources (Dunning and Kruger's preferred interpretation), leading to a non-linear relationship between *d*′ and metacognitive sensitivity. As discussed above, one-shot ratings are unable to disentangle bias, sensitivity and efficiency. Instead, by collecting trial-by-trial metacognitive judgments and calculating efficiency, it may be possible to ask whether efficiency itself is reduced in subjects with poorer skill.

## Implications of bias, sensitivity, and efficiency for studies of conscious awareness

There has been a recent interest in interpreting metacognitive measures as reflecting conscious awareness or subjective (often visual) phenomenological experience, and in this final section we discuss some caveats associated with these thorny issues. As early as Peirce and Jastrow (1885) it has been suggested that a subject's confidence can be used to indicate level of sensory awareness. Namely, if in making a perceptual judgment, a subject has zero confidence and feels that a pure guess has been made, then presumably the subject is not aware of sensory information driving the decision. If their judgment turns out to be correct, it would seem likely to be a fluke or due to unconscious processing.

However, confidence is typically correlated with task accuracy (type 1 *d*′)—indeed, this is the essence of metacognitive sensitivity. It has been argued that type 1 *d*′ itself should not be taken as a measure of awareness because unconscious processing may also drive type 1 *d*′ (Lau, 2008), as demonstrated in clinical cases such as blindsight (Weiskrantz et al., 1974). Lau (2008) gives further arguments as to why type 1 *d*′ is a poor measure of subjective awareness and argues that it should be treated as a potential confound. In other words, because type 1 *d*′ does not necessarily reflect awareness, in measuring awareness we should compare conditions where type 1 *d*′ is matched or otherwise controlled for. Importantly, to match type 1 *d*′, it is difficult to focus the analysis at a single-trial level, because *d*′ is a property of a task condition or group of trials. Therefore, Lau and Passingham (2006) created task conditions that were matched for type 1 *d*′ but differed in level of subjective awareness, permitting an analysis of neural activity correlated with visual awareness but not performance. Essentially, such differences between conditions reflect a difference in metacognitive bias despite type 1 *d*′ being matched.

In contrast, other studies have focused on metacognitive sensitivity, rather than bias, as a relevant measure of awareness. For instance, Kolb and Braun (1995) used binocular presentation and motion patterns to create stimuli in which subjects had positive type 1 *d*′ (in a localization task), but near-zero metacognitive sensitivity. Although this finding has proven difficult to replicate (Morgan and Mason, 1997), here we focus on the conceptual basis of their argument. The notion of taking a lack of metacognitive sensitivity as reflecting lack of awareness has also been discussed in the literature on implicit learning (Dienes, 2008), and is intuitively appealing. Lack of metacognitive sensitivity indicates that the subject has no ability to introspect upon the effectiveness of their performance. One plausible reason for this lack of ability is an absence of conscious experience on which the subject can introspect.

However, there is another possibility. Metacognitive sensitivity is calculated with reference to the external world (whether a judgment is objectively correct or incorrect), not the subject's experience, which is unknown to the experimenter. Thus, while low metacognitive sensitivity could be due to an absence of conscious experience, it could also be due to hallucinations, such that the subject vividly sees a false target and thus generates an incorrect type 1 response. Because of the vividness of the hallucination, the subject may reasonably express high confidence (a type 2 false alarm, from the point of view of the experimenter). In the case of hallucinations, the conscious experience does not correspond to objects in the real world, but it is a conscious experience all the same. Thus, low metacognitive sensitivity cannot be taken unequivocally to mean lack of conscious experience.

That said, we acknowledge the close relationship between metacognitive sensitivity and awareness in standard laboratory experiments in the absence of psychosis. Intuitively, metacognitive sensitivity is what gives confidence ratings their meaning. Confidence or bias fluctuates across individual trials (a single trial might be rated as “seen” or highly confident), whereas metacognitive sensitivity is a property of the individual, or at least a particular condition in the experiment. High confidence is only meaningfully interpretable as successful recognition of one's own effective processing when it can be shown that there is some reasonable level of metacognitive sensitivity; i.e., that confidence ratings were not given randomly. For instance, Schwiedrzik et al. (2011) used this logic to argue that differences in metacognitive bias reflected genuine differences in awareness, because metacognitive sensitivity was positive and unchanged in their experiment.

We note that criticisms also apply to using metacognitive bias to index awareness. In all cases, we would need to make sure that type 1 *d*′ is not a confound, and that the confidence level expressed is solely due to introspection of the conscious experience in question. Thus, the strongest argument for preferring metacognitive bias rather than metacognitive sensitivity as a measure of awareness is a conceptual one. Metacognitive sensitivity measures the ability of the subject to introspect, not what or how much conscious experience is being introspected upon on any given trial. For instance, in what is sometimes called type 2 blindsight, patients may develop a “hunch” that the stimulus is presented, without acknowledging the existence of a corresponding visual conscious experience. Such a hunch may drive above-chance metacognitive sensitivity (Persaud et al., 2011). More generally, it is unfortunate that researchers often prefer sensitivity or sensitivity measures simply because they are “bias free.” This advantage is only relevant when we have good reasons to want to exclude the influence of bias! Otherwise, bias and sensitivity measures are just different measures. This is true for both type 1 and type 2 analyses. Instead it might be useful to think of metacognitive sensitivity as a background against which awareness reports should be referenced. Metacognitive sensitivity indexes the amount we can trust the subject to tell us something about the objective features of the stimulus. But lack of trust does not immediately rule out an idiosyncratic conscious experience divorced from features of the world proscribed by the experimenter.

## Conclusions

Here we have reviewed measures of metacognitive sensitivity, and pointed out that bias is a confounding factor for popular measures of association such as gamma and phi. We point out that there are alternative measures available based on SDT and ROC analysis that are bias-free, and we relate these quantities to the calibration and resolution measures developed in the probability estimation literature. We strongly urge the application of the bias-free measures of metacognitive sensitivity reviewed above in future studies of metacognition. We distinguished between the related concepts of metacognitive bias (a difference in subjective confidence despite basic task performance remaining constant), metacognitive sensitivity (how good one is at distinguishing between one's own correct and incorrect judgments) and metacognitive efficiency (a subject's level of metacognition given a certain basic task performance or signal processing capacity). Finally, we discussed how these three concepts pose interesting questions for future studies of metacognition, and provide some cautionary warnings for directly equating metacognitive sensitivity with awareness. Instead, we advocate a more traditional approach that takes metacognitive bias as reflecting levels of awareness and metacognitive sensitivity as a background against which other measures should be referenced.

### Conflict of interest statement

The Editor Dr. Harriet Brown declares that despite having previously collaborated with the author Dr. Klaas Stephan the review process was handled objectively. The authors declare that the research was conducted in the absence of any commercial or financial relationships that could be construed as a potential conflict of interest.

## Footnotes

^1^These ratings may be elicited either prospectively or retrospectively.

^2^Another way of stating this is that phi is “margin sensitive”—the value of phi is affected by the marginal counts of Table 1 (the row and column sums) that describe an individual's task performance and bias.

^3^Kunimoto and colleagues labeled their type 2 *d*′ measure *a*′.
