# Supplementary material for: How to measure metacognition
Source: Front Hum Neurosci. 2014 Jul 15;8:443. doi: 10.3389/fnhum.2014.00443 (PMC4097944; doi:10.3389/fnhum.2014.00443)
Supplement: Supplementary file 1 [file DataSheet1.DOCX]

**APPENDIX**

Matlab code for calculating the area under the type 2 ROC.

function auroc2 = type2roc(correct, conf, Nratings)

% function auroc2 = type2roc(correct, conf, Nratings)

%

% Calculate area under type 2 ROC

%

% correct - vector of 1 x ntrials, 0 for error, 1 for correct

% conf - vector of 1 x ntrials of confidence ratings taking values 1:Nratings

% Nratings - how many confidence levels available

i = Nratings+1;

for c = 1:Nratings

H2(i-1) = length(find(conf == c & correct)) + 0.5;

FA2(i-1) = length(find(conf == c & ~correct)) + 0.5;

i = i-1;

end

H2 = H2./sum(H2);

FA2 = FA2./sum(FA2);

cum_H2 = [0 cumsum(H2)];

cum_FA2 = [0 cumsum(FA2)];

i=1;

for c = 1:Nratings

k(i) = (cum_H2(c+1) - cum_FA2(c))^2 - (cum_H2(c) - cum_FA2(c+1))^2;

i = i+1;

end

auroc2 = 0.5 + 0.25*sum(k);
